# *Toxoplasma* infection in pregnant women: a current status in Songklanagarind hospital, southern Thailand

**DOI:** 10.1186/1756-3305-7-239

**Published:** 2014-05-22

**Authors:** Hemah Andiappan, Veeranoot Nissapatorn, Nongyao Sawangjaroen, Waenurama Chemoh, Yee Ling Lau, Thulasi Kumar, Subashini Onichandran, Chitkasaem Suwanrath, Verapol Chandeying

**Affiliations:** 1 Department of Parasitology, Faculty of Medicine, University of Malaya, Kuala Lumpur 50603, Malaysia; 2 Department of Microbiology, Faculty of Science, Prince of Songkla University, Hat Yai, Songkhla 90112, Thailand; 3 Department of Obstetrics and Gynecology, Faculty of Medicine, Prince of Songkla University, Hat Yai, Songkhla 90112, Thailand; 4 Faculty of Medicine, University of Phayao, Phayao 56000, Thailand

**Keywords:** Toxoplasmosis, *Toxoplasma gondii*, Seroprevalence, Risk factors, Pregnant women, Thailand

## Abstract

**Background:**

Toxoplasmosis, being one of the TORCH’s infections in pregnant women, is caused by *Toxoplasma gondii*, an obligate intracellular protozoan parasite. This parasitic infection in pregnancy congenitally causes severe outcomes to their fetus and newborn. This study aimed to determine the seroprevalence and stages of *Toxoplasma* infection in pregnant women and its associated risks exposures.

**Methods:**

The study was conducted within the pregnant women attending the antenatal clinic (ANC) at Songklanagarind hospital, Hat Yai, Songkhla province, Thailand. The sera of a total of 760 consecutive pregnant women were screened using standard commercial ELISA kits for detection of anti-*Toxoplasma* IgG and IgM antibodies. IgG avidity in the seropositive for both anti-*Toxoplasma* IgG and IgM antibodies were also assessed. The pregnant women’s socio-demographic, obstetrics and risk factors associated with *Toxoplasma* seropositivity data were analyzed using univariate and multivariate analyses.

**Results:**

From the total 760 pregnant women, 190 (25%, 95% CI = 22.05-28.20) were positive for anti-*Toxoplasma* antibodies. Of these, 167 (22.0%, 95% CI = 19.0-25.0) were positive for only anti-*Toxoplasma* IgG antibody and 23 (3.0%, 95% CI = 2.0-4.0) were positive for both anti-*Toxoplasma* IgG and IgM antibodies. All these samples were high avidity, indicated the infection occured prior to four to five months. By applying statistical univariate analysis, age group, occupation and sources of drinking water showed a significant association with *Toxoplasma* seropositivity (*p* < 0.05). Multivariate logistic regression analysis further indicated that the significant factors associated with *Toxoplasma* seropositivity are age ≥26 (OR = 1.65, 95% CI = 1.11-2.44), working as laborer (OR = 1.57, 95% CI = 1.13-2.18) and drinking unclean (piped/tap/rain) water (OR = 1.75, 95% CI = 1.08-2.84).

**Conclusion:**

The pregnant women in the active age group, working as laborers and exposure to unclean drinking water from various sources were at higher risk of *Toxoplasma* infection. Therefore, health education and the awareness of risk exposures regarding this parasitic disease are required to minimize the effects of this parasitic infection in pregnant women as well as in the general population.

## Background

*Toxoplasma gondii*, an obligate intracellular protozoan parasite [1], is capable of causing severe and life threatening conditions in pregnant women and immunocompromised individuals. The sources of this parasitic infection are by the ingestion of raw and/or undercooked meat containing parasite cysts in the animal tissues, by consuming oocysts infected water and/or food, or having contact with cat fecal contaminated soil [2]. *Toxoplasma* infection in pregnant women, poses great concern to the fetus. Severe impairment occurs internally, i.e. hydrocephalus, intracerebral calcification, retinochoroiditis and mental retardation, however, clinical presentation in the newborn is asymptomatic at birth in general [3].

Detection of anti-*Toxoplasma* antibodies in pregnant women is the most widely used approach in diagnosis of this parasitic infection [4]. The detected antibodies, which indicate recent or past infection in the pregnant women, are important to confirm whether the fetus is at risk [5]. The presence of anti-*Toxoplasma* IgG antibodies represents past infection meanwhile the detection of anti-*Toxoplasma* IgM antibodies indicates recent infection [6]. However, the specified IgM antibodies remain for several months or years after initial infection [7]. This limitation causes a problem in diagnosis of whether the maternal infection occurs prior to or after conception. Misinterpretation of IgM positive results in conventional single-serum assay may lead to misdirection in treatment and termination of pregnancy [8]. The assessment of IgG avidity for *Toxoplasma* infection in pregnant women has been introduced in recent studies to assist in discrimination between past and recently acquired infection. The result of this avidity test is most helpful in determining the infection of *Toxoplasma* in pregnant woman, especially for those who are in their first trimester [9].

Therefore, this study was conducted to determine the current seroprevalence of *Toxoplasma* infection among pregnant women attending the ANC at Songklanagarind hospital, southern Thailand, to investigate the association between plausible risk factors of *Toxoplasma* infection with the seropositive pregnant women and to validate the stages of *Toxoplasma* infection in these pregnant women using avidity measurement.

## Methods

### Study site and population

A prospective cross-sectional study was carried out at the ANC of Songklanagarind hospital, Hat Yai, Songkhla province, Thailand from December 2012 to August 2013. This public hospital attached to Prince of Songkla University, with its capacity of 850 in-patient beds, is located in the south of Thailand and was built to facilitate the teaching, research, and training for medical personnel in various disciplines, and for the provision of healthcare to the general public, particularly among Southern Thais. The study included 760 eligible pregnant women who gave informed consent before this study. The questionnaire [10] was designed to detect socio-demographic and biologically plausible risk factors associated with *Toxoplasma* infection, and clinical history and presenting signs and symptoms relating to toxoplasmosis (if any). This study was conducted with the approval from the ethical committee of the Faculty of Medicine, Prince of Songkla University, Thailand (Ethics number: EC 52-268-12-1-3).

### Serum collection

Approximately 5 mL of venous blood samples were drawn and their sera were collected and kept at −20°C until further testing.

### Screening for anti-*Toxoplasma* IgG and IgM antibodies

Anti-*Toxoplasma* IgG and IgM antibodies were screened by using a standard ELISA commercial kit (IgG-Trinity Biotech and IgM-Trinity Biotech, New York) in accordance with the manufacturer's instructions. A positive sample for the anti-*Toxoplasma* IgG and IgM antibodies was also tested for its avidity using a standard ELISA commercial kit (IgG-NovaLisa Dietzenbach, Germany); high avidity (>40%) indicated a past infection (of at least 4–5 months) and a low avidity (<40%) indicated a recently acquired infection (within 4–5 months).

### Statistical analysis

Data obtained from both the questionnaire and laboratory tests were entered, edited, and analyzed using the statistical software SPSS version 17.0 (SPSS, Inc., Chicago, IL). The data with quantitative variables were expressed as the mean (±SD) and range, whereas qualitative variables were estimated and presented as frequencies and percentages. Univariate analyses and the 2 test were used to investigate the association between *Toxoplasma* seropositivity as a dependent variable and possible demographic and risk factors as independent variables; *p* < 0.05 was regarded as being statistically significant.

## Results

A total of 760 pregnant women were recruited. Their age range was from 14 to 47 years with a mean of 29.5 ± 6.34 years. The majority of these women was between 26 and 35 years (408, 53.7%), had tertiary education (369, 48.6%) and worked as laborers (317, 41.7%). Most of these pregnant women were in their first trimester of pregnancy (621, 81.7%), had no history of receiving antibiotic treatment (664, 87.4%), have one or no child (637, 83.8%) and had no experience of miscarriage (592, 77.9%) as shown in Table 1.

**Table 1:** **Socio-demographic, obstetric profiles and risk factors associated with *Toxoplasma *** **seropositivity in pregnant women by univariate analysis**

**Variable**	**No. of pregnant women**		***P-value***
	**Total (n, %)**	***Toxoplasma*** **seropositive (n, %)**	
	**N = 760**	**N = 190**	
**Demographic profile**			
**Age**			
Range 14–47 years			
Mean 29.5 ± 6.34 years			
**Age group**			0.042
14–25	214 (28.2)	40 (18.7)	
26–35	408 (53.7)	112 (27.5)	
36-47	138 (18.2)	38 (27.5)	
**Education**			0.311
Primary	48 (6.3)	16 (33.3)	
Secondary	343 (45.1)	80 (23.3)	
Tertiary	369 (48.6)	94 (25.5)	
**Occupation**			0.002
Labourer	317 (41.7)	95 (29.9)	
Non-Labourer	275 (36.2)	69 (25.0)	
Unemployed	168 (22.1)	26 (15.5)	
**Obstetric history**			
**Antibiotic usage**			0.801
No	664 (87.4)	167 (25.2)	
Yes	96 (12.6)	23 (23.9)	
**Trimester**			
First	621 (81.7)	162 (26.1)	0.255
Second	129 (17.0)	27 (20.9)	
Third	10 (1.3)	1 (10.0)	
**No. children**			0.460
≤1	637 (83.8)	156 (24.5)	
≥2	123 (16.2)	34 (27.6)	
**History of abortion**			1.000
No	592 (77.9)	148 (25.0)	
Yes	168 (22.1)	42 (25.0)	
**Awareness of toxoplasmosis**			0.320
No	708 (93.2)	180 (25.4)	
Yes	52 (6.8)	10 (19.2)	
**Risk factors of toxoplasmosis**			
**Close contact with cats**			0.258
No	482 (63.4)	114 (23.7)	
Yes	278 (36.6)	76 (27.3)	
**Consumption of undercooked meat**			0.408
No	275 (36.2)	64 (23.3)	
Yes	485 (63.8)	126 (25.9)	
**Receiving blood transfusion**			0.414
No	758 (99.7)	190 (25.1)	
Yes	2 (0.26)	0 (0)	
**Drinking water from various source**			0.013
Boiled	75 (9.8)	24 (32.0)	
Mineral/Filtered	597 (78.6)	135 (22.6)	
Piped/tap/rain	88 (11.6)	31 (35.2)	
**Drinking milk**			0.693
No	97 (12.8)	27 (27.8)	
Boiled	8 (1.1)	3 (37.5)	
Pasteurized	649 (85.4)	158 (24.3)	
Non-Pasteurized	6 (0.8)	2 (33.3)	
**Having contact with soil**			0.395
No	316 (41.6)	74 (23.4)	
Yes	444 (58.4)	116 (26.1)	
**Close contact with other animals**			0.967
No	369 (48.6)	92 (24.9)	
Yes	391 (51.4)	98 (25.0)	

Overall, the seroprevalence of *Toxoplasma* infection in our study was 190 (25.0%, 95% CI = 22.05-28.20) of which 167 (22.0%, 95% CI = 19.0-25.0) were positive for only anti-*Toxoplasma* IgG antibodies and 23 (3.0%, 95% CI = 2.0-4.0) were positive for both anti-*Toxoplasma* IgG and IgM antibodies. Serum samples positive for only anti-*Toxoplasma* IgM antibodies (19, 2.5%) were re-screened by collecting second serum samples after intervals of four weeks apart. These serum samples were later reported as false positive since there was no sero-conversion found.

The 23 samples that were seropositive for both anti-*Toxoplasma* IgG and IgM antibodies were subsequently assessed by IgG avidity test. All these samples showed high avidity, indicating they may have acquired the infection four to five months earlier. It was noted that 20 of these samples were in their first trimester and the remaining three were in their second trimester of pregnancy.

For univariate analysis, this study verified that age group, occupation and source of drinking water had significant associations with seropositive pregnant women (*p* < 0.05), as shown in Table 1. After the multivariate logistic regression analysis was performed, it was confirmed that age ≥26 (OR = 1.65, 95% CI = 1.11-2.44), working as a laborer (OR = 1.57, 95% CI = 1.13-2.18) and drinking unclean (piped/tap/rain) water (OR = 1.75, 95% CI = 1.08-2.84) were identified as significant risk factors for *Toxoplasma* acquisition, as shown in Table 2.

**Table 2:** **Multivariate logistic regression analysis for demographic profiles and risk factors associated with *Toxoplasma *** **seropositive pregnant women**

**Variable**	**Adjusted odds ratio**	**95% CI**		***P*** **value**
Age ≥26	1.65	1.11	2.44	0.012
Working as labourer	1.57	1.13	2.18	0.007
Drinking Piped/tap/rain water	1.75	1.08	2.84	0.018

Adjusted variables include age group, occupation and source of drinking water in this statistical analysis.

## Discussion

*Toxoplasma* infection in pregnant women shows variation in seroprevalence globally. From our findings, the overall seroprevalence of chronic *Toxoplasma* infection was 25% in the recruited Thai pregnant women. This finding fell within the range of 2.6% - 28% of seroprevalence that have been reported in Thailand [10-18]. However, our prevalence rate is still higher compared to previous studies conducted recently in our Asian counterparts such as China, Japan and Taiwan being 3.98%, 10.3% and 11.8%, respectively [19-21]. Globally, the seroprevalence of *Toxoplasma* infection remains high in many countries across the continents, e.g., 84.7% in Congo [22], 83.6% in Ethiopia [23], 45% in India [24], 59% in Brazil [25] and 30.9% in Tanzania [26]. The seroprevalence may vary in a global view, but the risk of this parasitic infection in human populations, especially in pregnant women, still holds a great interest. Our findings imply that we need to take a holistic approach to educate our pregnant women on toxoplasmosis in order to reduce the infection rate and the overall disease burden in our society.

In this study, 2.5% of pregnant women were false positive for anti-*Toxoplasma* IgM antibodies after re-screening their second serum sample collected four weeks later to test for seroconversion. This phenomenon could be due to host natural IgM antibodies reacting with *Toxoplasma* antigens without infection [1,8]. Meanwhile, the pregnant women who were positive for both anti-*Toxoplasma* IgG and IgM antibodies have high IgG avidity, indication of past infection. The specified IgM antibodies may remain for several months or years after initial infection [7]. The majority of these pregnant women were in their first trimester of pregnancy. This is of great concern as the effect of *Toxoplasma* infection is more severe to the fetus during the first trimester of pregnancy [27]. In the incidence of positive for both anti-*Toxoplasma* IgG and IgM antibodies, possibilities of acute infection or false IgM positive results were predicted. IgG avidity measurement assists as a confirmatory tool in the determination of infection stages in the suspected pregnant women with the availability of a single serum sample [28]. A high avidity of IgG antibodies indicates that there is no risk for congenital toxoplasmosis in the fetus, especially for pregnant women in their first trimester, regardless of the IgM antibodies results [5].

Our epidemiological data showed that age ≥ 26 years, working as a laborer and drinking unclean water were significant factors associated with *Toxoplasma* infection. This could be generally explained that the seroprevalence of *Toxoplasma* infection increases by age [29] and also specifically by their low-socioeconomic and poor hygiene practice which can play as an important role in the transmission of the parasites [30-32].

Pregnant women with more than one child have been shown a significant association with *Toxoplasma* infection [33] and this could be due to lack of cleanliness among their children [34]. However, no significant association was found between obstetric histories and *Toxoplasma* seropositivity in our study. Most of these pregnant women were not aware of toxoplasmosis and this could lead to high *Toxoplasma* seropositivity, as they do not know how to protect themselves from this parasitic infection [35,36].

Concerning other plausible risk factors, some of our pregnant women had a history of close contacts with cats (37%) but had no significant association with *Toxoplasma* infection. This finding is, however, contrary to a few of the previous studies reported, where having close contact with felines was shown as one of the vital factors in transmission of *Toxoplasma* infection [37,38]. Studies have also shown that *Toxoplasma* infection can be transmitted by other animals to their owners [39-41]. *Toxoplasma* cysts infected animals’ tissues for human consumption is a mode of disease transmission [42-44]. Most of our pregnant women had consumed undercooked meat but there is no significant association with high *Toxoplasma* infection. This could be due to absence of contamination by *Toxoplasma* cysts in the consumed meat and needs to be further studied. Our finding is consistent with a previous study reported in this region [11], showing that drinking unclean water (pipe/tap/rain) had significant association with *Toxoplasma* seropositive pregnant women, indicating the water may be contaminated with *Toxoplasma* oocysts. However, high seropositive (32%) were also found in pregnant women who consumed boiled water but were not statistically significant. Based on the above results, this could be a good indicator of other confounding factors contributing to *Toxoplasma* infection and requires further studies.

## Conclusion

Our findings showed high *Toxoplasma* infection rates in this group of pregnant women and showed significantly higher risk with age group, low socioeconomic status and drinking unclean water. The following guidelines should be implemented to eliminate *Toxoplasma* infection and eventually eradicate its disease burden; Firstly, a routine screening for toxoplasmosis among women in the reproductive age group and pregnant women, especially for those in their early pregnancy is strongly encouraged for monitoring and preventive purposes. Secondly, health education on toxoplasmosis (brochures were attached as Additional file 1) and its risk exposures is required to increase the awareness about this disease and to minimize the effects of this *Toxoplasma* infection in the general population and pregnant women in particular. Lastly, serological diagnosis through the detection of anti-*Toxoplasma* antibodies and IgG avidity measurement in pregnant women assist the dating of the infection and as well as determining the decision for course of treatment especially those in their early pregnancies.

## Competing interest

All authors declare having no conflicts of interest.

## Authors’ contributions

All authors were involved in the design of the study. VN was the principal investigator. VN and NS drafted the protocol, consent forms, and paper with input from the other authors. HA, TK, SO and WC were responsible for sample collection, serological analysis and data analysis. HA and WC were responsible for statistical analysis and interpretation. LYL, NS, CS, VC and VN were involved in data analysis, statistical analysis and interpretation and results discussion and inputs. All authors contributed to the writing of the paper and approved the final version.

## Supplementary Material

Additional file 1 **Health care education on Toxoplasmosis in pregnant women.** Brochures in English Language. Brochures in Thai language. Click here for file
