# Supplementary material for: Toxoplasma infection in pregnant women: a current status in Songklanagarind hospital, southern Thailand
Source: Parasit Vectors. 2014 May 22;7:239. doi: 10.1186/1756-3305-7-239 (PMC4035750; doi:10.1186/1756-3305-7-239)

Supplement 1: Health care education on Toxoplasmosis in pregnant women.

Brochures in English Language.


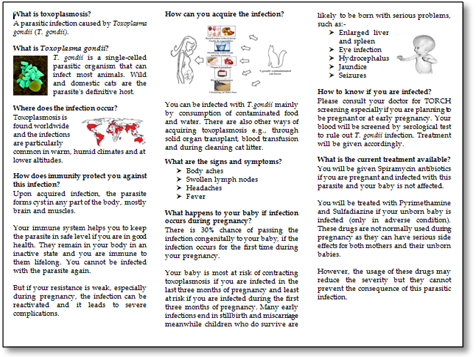

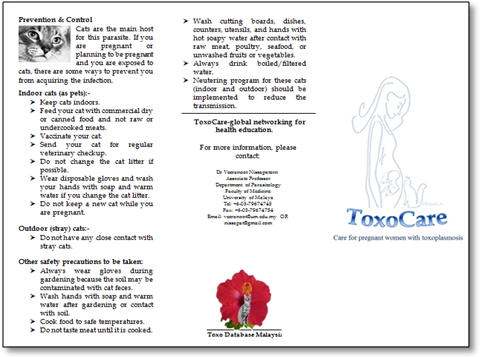


Brochures in Thai language.


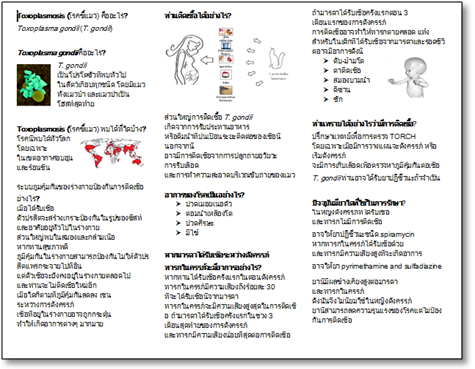

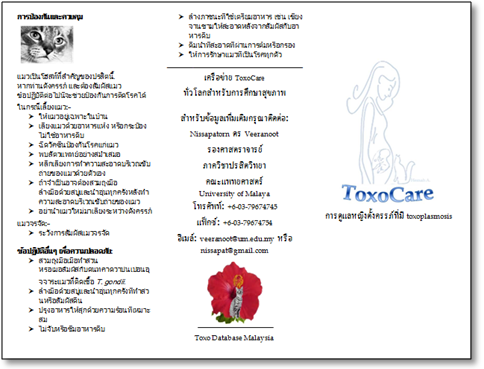

Supplement: Additional file 1 — Health care education on Toxoplasmosis in pregnant women. Brochures in English Language. Brochures in Thai language. [file 1756-3305-7-239-S1.doc]
